# Association between plain water intake and the risk of osteoporosis among middle-aged and elderly people in the United States: a cross-sectional study

**DOI:** 10.3389/fnut.2025.1527771

**Published:** 2025-03-18

**Authors:** Xudong Wang, Meng Wang, Zijian Guo, Chuan Xiang

**Affiliations:** ^1^Department of Orthopedics, The Second Hospital of Shanxi Medical University, Taiyuan, Shanxi, China; ^2^Academy of Medical Sciences, Shanxi Medical University, Taiyuan, Shanxi, China

**Keywords:** plain water intake, osteoporosis, middle-aged and elderly people, cross-sectional study, National Health and Nutrition Examination Survey

## Abstract

**Background:**

The connection between plain water intake (PWI) and osteoporosis risk is still unclear. The investigation aimed to identify the relationship between PWI and osteoporosis risk in middle-aged and elderly individuals in the United States (US).

**Methods:**

This cross-sectional study was conducted among participants aged 50 years and older in the following waves of the National Health and Nutrition Examination Survey (NHANES): 2007–2008, 2009–2010, 2013–2014, and 2017–2018. The relationship between PWI and osteoporosis risk was examined by multivariable logistic regression models, accompanied by subgroup analyses and interaction tests. Smooth curve fitting and threshold effect analysis were utilized.

**Results:**

The present investigation included 6,686 participants. In accordance with the fully adjusted model, individuals in the highest PWI tertile had a significantly reduced risk of osteoporosis in contrast to those in the lowest tertile [odds ratio (OR) = 0.62; 95% confidence interval (CI): 0.49–0.77; *P* for trend<0.001]. After adjusting for all covariates, a higher PWI was linked to a decreased risk of osteoporosis (OR = 0.92; 95% CI: 0.86–0.98; *p* = 0.008). No significant interactions were detected in the subgroup analyses for age, gender, race, body mass index, diabetic history, hypertension status, smoking history, consumption of prednisone or cortisone, or moderate or strenuous activity (all *P* for interaction>0.05). Smooth curve fitting and threshold effect analysis revealed that when PWI was less than 1,220 mL/day, there was a significant negative connection between PWI and osteoporosis risk (OR = 0.79; 95% CI: 0.70–0.89; *p* < 0.001); nevertheless that association was not significant when PWI was greater than 1,220 mL/day (OR = 1.06; 95% CI: 0.95–1.17; *p* = 0.288).

**Conclusion:**

The outcomes of our investigation indicated that among middle-aged and older US adults, a higher PWI was connected with a moderately reduced osteoporosis risk. Managing PWI might reduce the osteoporosis risk.

## Introduction

Osteoporosis is a systemic skeletal disorder defined by diminished bone mineral density (BMD) and microarchitectural degradation of bone tissue (1–3). With the advanced aging of the population, osteoporosis has emerged as the most prevalent bone metabolic disorder (4). Almost 14.1 million individuals aged 50 and older suffer from osteoporosis in the United States (US), and the incidence rate exhibits a steady increase (5–7). Osteoporosis can result in higher fragility of the bone and an elevated fracture risk, which impacts almost all skeletal sites due to the systemic nature of the disease (1, 3, 8, 9). Traditionally, hip and vertebral fractures have been regarded as prototypical osteoporotic fractures (1). However, a far greater incidence of osteoporotic fractures has been observed at all other sites (i.e., excluding the hip and vertebrae) (10). The consequences of osteoporotic fractures include serious complications, reduced quality of life, elevated disabilities, and raised death rates (11). Moreover, osteoporosis and its associated fractures impose an enormous financial burden on patients, their families, and society (12–14). Therefore, preventing osteoporosis is vital.

Diet has a vital role in modifying the risk of osteoporosis and contributing to its prevention (15). Water is an important nutrient in the diet and is connected with several physiological functions, such as metabolism, modulation of body temperature, transportation of nutrients, and elimination of waste products (16–18). There are various sources of water consumed in daily life, including tea, coffee, sugar-sweetened beverages, and plain water. The source of water consumed is important for bone health. Huang et al. discovered that consuming tea provided a protective effect against osteoporosis, especially in women and middle-aged adults (19). Xu et al. (20) reported that regular moderate consumption of coffee may provide protection against osteoporosis between older US adults and those in middle age. Notably, a systematic review and meta-analysis comprising 26 publications exhibited that the intake of beverages that were sweetened by sugar was negatively related to BMD in adults (21). Nevertheless, few investigations have focused on the connection between plain water intake (PWI) and osteoporosis risk.

Therefore, this cross-sectional study was performed to determine the link between PWI and osteoporosis risk among older US adults and those in middle age.

## Materials and methods

### Study population

The nutritional and health status of the US people were evaluated utilizing the National Health and Nutrition Examination Survey (NHANES), which is a large-scale cross-sectional study executed by the National Center for Health Statistics (NCHS). Information on diet, demographics, questionnaires, examinations, and laboratories has been published every 2 years. The Institutional Review Board of the NCHS authorized the entire program, and every individual signed an informed consent form.

**Figure 1:**
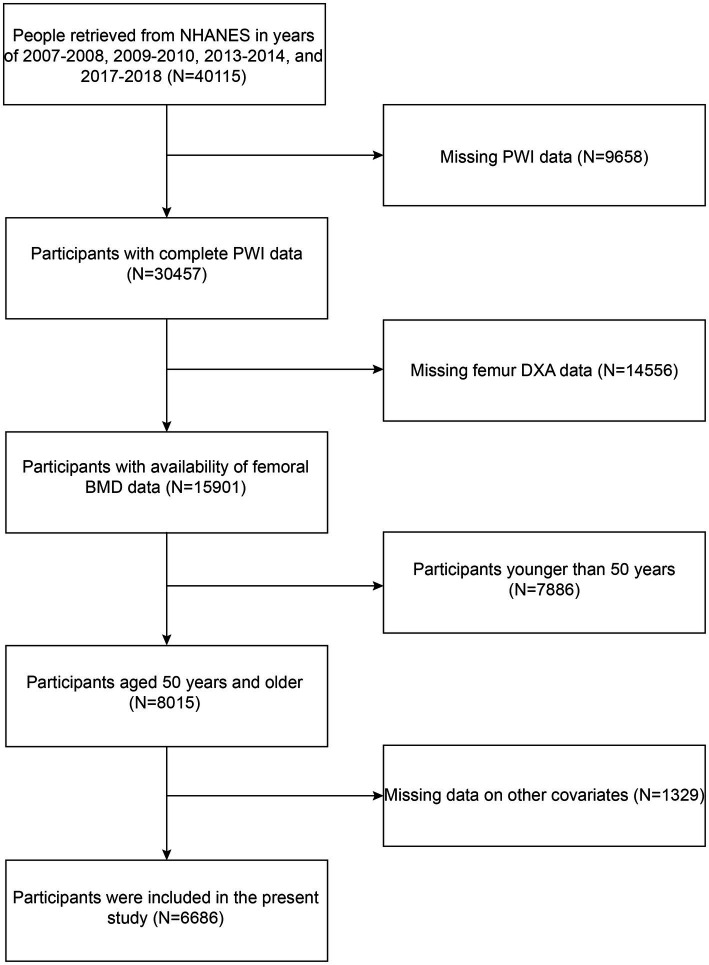
Flow chart of the inclusion and exclusion criteria.

All of the information in this investigation was retrieved from the following NHANES cycles: 2007–2008, 2009–2010, 2013–2014, and 2017–2018. Because it was only during these cycles that femur dual-energy X-ray absorptiometry (DXA) data and information about vitamin D intake and dietary supplements were recorded. The criteria for participant inclusion in our investigation were as follows: (1) complete PWI data; (2) availability of femoral BMD data; and (3) aged 50 years and older. The criteria of exclusion were established as follows: (1) missing PWI data; (2) missing femur DXA data; (3) age younger than 50 years; and (4) missing data on other covariates. Firstly, data for 40,115 participants were chosen from the following NHANES cycles: 2007–2008, 2009–2010, 2013–2014, and 2017–2018. Subsequently, 9,658 participants were excluded owing to absent PWI information. Participants with missing information on femur DXA (*N* = 14,556) and those with age less than 50 years (*N* = 7,886) or with lost information on other covariates (*N* = 1,329) were also excluded. Ultimately, the present study comprised 6,686 participants (Figure 1).

### Measurement of PWI

PWI is known as the overall amount of water consumed over a 24-h timeframe, including bottled water, ordinary tap water, spring water, and water obtained from the consumption of fountains or water coolers. The 24-h PWI of each participant was collected via face-to-face interviews, and the information was subsequently collected via telephone interviews 3–10 days later. The present investigation utilized the mean of two recordings for statistical analysis to determine the long-term average PWI of the population in the US.

### Definition of osteoporosis

The BMD of the femoral region, as measured by DXA, was used to evaluate whether an individual was diagnosed with osteoporosis. Depending on the classification standards of the World Health Organization, osteoporosis was diagnosed when BMD measurements in any femoral region were greater than 2.5 standard deviations (SDs) below those of the reference group (young adults) (22). The current study examined the femoral BMD at the whole femur, neck of the femur, trochanter, and intertrochanter sites. The diagnostic thresholds were 0.68 g/cm^2^ for the whole femur, 0.59 g/cm^2^ for the femur neck, 0.49 g/cm^2^ for the trochanter, and 0.78 g/cm^2^ for the intertrochanter (23).

### Other covariates

Based on prior research and clinical experience, we collected data on covariates that may influence the connection between PWI and osteoporosis risk. The selected covariates were obtained from demographic, examination, questionnaire, laboratory and dietary data. The covariates extracted from demographic data included age, gender, race, level of education, marital status, and family poverty–income ratio (PIR). Body mass index (BMI) data were extracted from the examination information. The factors obtained from the questionnaire data consisted of diabetic history, hypertension status, thyroid disease, smoking history, consumption of prednisone or cortisone, milk product consumption, engagement in moderate or strenuous activity, and fracture. The covariates extracted from laboratory data comprised serum vitamin D, total calcium, alanine aminotransferase, asparate aminotransferase, creatinine, and uric acid. The covariates obtained from the dietary data included alcohol consumption, tea consumption, vitamin D supplementation, calcium supplementation, vitamin D intake, calcium intake, caffeine intake, energy intake, protein intake, and other liquid intake. Smoking history was ascertained by inquiring if individuals had consumed a minimum of 100 cigarettes throughout their lives. Moderate or strenuous activity was characterized as a minimum of ten continuous minutes of sports, fitness, or recreational activities that resulted in a minor or significant elevation in heart rate or breathing over the preceding 30 days or during a typical week.

### Statistical analysis

The R Version 3.4.3 (The R Foundation, http://www.R-project.org) and Empower (X&Y Solutions, Inc., Boston, MA, United States) programs were utilized to perform the statistical analysis. We employed proportions to provide a summary of categorical data and means ± SDs to characterize continuous variables. We utilized a chi-square test for categorical variables and a Student’s t-test for continuous variables in order to assess variations among patients classified by either the existence or absence of osteoporosis. The correlation between PWI and osteoporosis risk was examined by employing multivariable logistic regression models. Model 1 was unadjusted for covariates; Model 2 underwent adjustment for gender, age, and race; and Model 3 underwent adjustment for all covariates, encompassing age, gender, race, level of education, marital status, PIR, BMI, diabetic history, hypertension status, thyroid disease, smoking history, consumption of prednisone or cortisone, moderate or strenuous activity, fracture, milk product consumption, alcohol consumption, tea consumption, vitamin D supplementation, calcium supplementation, vitamin D intake, calcium intake, caffeine intake, serum vitamin D, total calcium, alanine aminotransferase, asparate aminotransferase, creatinine, uric acid, energy intake, protein intake, and other liquid intake. To strengthen the data analysis, we utilized each 500 mL/day PWI as a unit and classified PWI into three groups according to tertiles. The dependability of the regression analysis outcomes was improved utilizing a trend test. Furthermore, we conducted subgroup analyses and interaction tests for particular variables, including age, gender, race, BMI, diabetic history, hypertension status, smoking history, consumption of prednisone or cortisone, or moderate or strenuous activity, to explore heterogeneity across subgroups. Smooth curve fitting and threshold effect analysis were utilized to explore possible nonlinear connections between PWI and osteoporosis risk. A *p*-value below 0.05 was deemed statistically significant.

## Results

### Baseline features of the enrolled participants

Table 1 presents the baseline features of the recruited participants. There were 734 individuals in the osteoporosis group and 5,952 individuals in the nonosteoporosis group. Individuals without osteoporosis had a greater PWI (every 500 mL/day) than did those with osteoporosis (1.80 ± 1.63 vs. 1.49 ± 1.51, *p* < 0.001). Moreover, there were significant between-group variations in gender, race, age, level of education, PIR, marital status, BMI, thyroid disease, smoking history, consumption of prednisone or cortisone, moderate or strenuous activity, fracture, alcohol consumption, vitamin D supplementation, calcium supplementation, calcium intake, caffeine intake, serum vitamin D, alanine aminotransferase, creatinine, uric acid, energy intake, protein intake, and other liquid intake (*p* < 0.05). Nevertheless, no significant variation was detected in diabetic history, hypertension status, milk product consumption, tea consumption, vitamin D intake, total calcium, and asparate aminotransferase between both groups (*p* > 0.05).

**Table 1:** Baseline features of the enrolled participants.

Feature	Nonosteoporosis	Osteoporosis	*P*-value
No. of participants	5,952	734	
Age, *n* (%)			<0.001
< 65	3,342 (56.15%)	208 (28.34%)	
≥ 65	2,610 (43.85%)	526 (71.66%)	
Gender, *n* (%)			<0.001
Male	3,253 (54.65%)	158 (21.53%)	
Post-menopausal female	2,406 (40.42%)	546 (74.39%)	
Non-menopausal female	293 (4.92%)	30 (4.09%)	
Race, *n* (%)			<0.001
Hispanic	1,327 (22.30%)	118 (16.08%)	
Non-Hispanic White	2,984 (50.13%)	490 (66.76%)	
Non-Hispanic Black	1,200 (20.16%)	61 (8.31%)	
Other	441 (7.41%)	65 (8.86%)	
Level of education, *n* (%)			0.003
Less than high school	1,433 (24.08%)	192 (26.16%)	
High school	1,405 (23.61%)	205 (27.93%)	
More than high school	3,114 (52.32%)	337 (45.91%)	
Marital status, *n* (%)			<0.001
Married/Living with partner	3,817 (64.13%)	345 (47.00%)	
Widowed/Divorced/Separated	1750 (29.40%)	349 (47.55%)	
Never married	385 (6.47%)	40 (5.45%)	
PIR, *n* (%)			<0.001
≤ 1	909 (15.27%)	128 (17.44%)	
1–3	2,467 (41.45%)	370 (50.41%)	
> 3	2,576 (43.28%)	236 (32.15%)	
BMI, n (%)			<0.001
< 30	3,594 (60.38%)	606 (82.56%)	
≥ 30	2,358 (39.62%)	128 (17.44%)	
Diabetic history, *n* (%)			0.058
Yes	1,151 (19.34%)	125 (17.03%)	
No	4,572 (76.81%)	590 (80.38%)	
Borderline	229 (3.85%)	19 (2.59%)	
Hypertension status, *n* (%)			0.645
Yes	3,182 (53.46%)	399 (54.36%)	
No	2,770 (46.54%)	335 (45.64%)	
Thyroid disease, *n* (%)			<0.001
Yes	844 (14.18%)	166 (22.62%)	
No	5,108 (85.82%)	568 (77.38%)	
Smoking history, *n* (%)			0.041
Yes	3,035 (50.99%)	345 (47.00%)	
No	2,917 (49.01%)	389 (53.00%)	
Consumption of prednisone or cortisone, *n* (%)			0.009
Yes	355 (5.96%)	62 (8.45%)	
No	5,597 (94.04%)	672 (91.55%)	
Moderate or strenuous activity, *n* (%)			<0.001
Yes	2,662 (44.72%)	250 (34.06%)	
No	3,290 (55.28%)	484 (65.94%)	
Fracture, *n* (%)			<0.001
Yes	698 (11.73%)	167 (22.75%)	
No	5,254 (88.27%)	567 (77.25%)	
Milk product consumption, *n* (%)			0.250
Yes	4,856 (81.59%)	586 (79.84%)	
No	1,096 (18.41%)	148 (20.16%)	
Alcohol consumption, *n* (%)			<0.001
Yes	1747 (29.35%)	159 (21.66%)	
No	4,205 (70.65%)	575 (78.34%)	
Tea consumption, *n* (%)			0.256
Yes	2,310 (38.81%)	269 (36.65%)	
No	3,642 (61.19%)	465 (63.35%)	
Vitamin D supplementation, *n* (%)			0.003
Yes	2,736 (45.97%)	380 (51.77%)	
No	3,216 (54.03%)	354 (48.23%)	
Calcium supplementation, *n* (%)			<0.001
Yes	2,830 (47.55%)	397 (54.09%)	
No	3,122 (52.45%)	337 (45.91%)	
Vitamin D intake (μg/day, mean ± SDs)	4.68 ± 4.40	4.61 ± 4.45	0.748
Calcium intake (mg/day, mean ± SDs)	868.49 ± 451.09	812.56 ± 432.28	<0.001
Caffeine intake, (mg/day, mean ± SDs)	164.08 ± 182.88	138.75 ± 152.92	<0.001
Serum vitamin D (nmol/L, mean ± SDs)	70.79 ± 28.81	76.17 ± 33.67	<0.001
Total calcium (mg/dL, mean ± SDs)	9.42 ± 0.38	9.43 ± 0.41	0.341
Alanine aminotransferase (U/L, mean ± SDs)	24.25 ± 18.95	20.19 ± 11.35	<0.001
Asparate aminotransferase (U/L, mean ± SDs)	25.45 ± 13.83	25.15 ± 14.48	0.576
Creatinine (mg/dL, mean ± SDs)	0.97 ± 0.46	0.96 ± 0.57	<0.001
Uric acid (mg/dL, mean ± SDs)	5.69 ± 1.41	5.25 ± 1.49	<0.001
Energy intake (kcal/day, mean ± SDs)	1922.26 ± 749.85	1690.95 ± 680.17	<0.001
Protein intake (g/day, mean ± SDs)	76.47 ± 32.33	65.29 ± 28.03	<0.001
PWI (every 500 mL/day, mean ± SDs)	1.80 ± 1.63	1.49 ± 1.51	<0.001
Other liquid intake (ml/day, mean ± SDs)	1746.25 ± 802.85	1533.24 ± 668.69	<0.001

PWI, plain water intake; PIR, family poverty–income ratio; BMI, body mass index; SDs, standard deviations.

### Associations between PWI and the risk of osteoporosis

The associations between PWI and osteoporosis risk are shown in Table 2. PWI was altered from a continuous variable into a categorical variable according to tertiles. According to Model 1 (not adjusted for covariates), participants in the greatest PWI tertile group had a 44% reduced risk of osteoporosis in contrast to those in the group of the lowest PWI tertile [odds ratio (OR) = 0.56; 95% confidence interval (CI): 0.47–0.68; *P* for trend<0.001]. Similarly, participants in the group of the greatest PWI tertile had a significantly lower risk of osteoporosis in contrast to those in the group of the lowest PWI tertile, as shown by Model 2 (adjusted for the main covariates; OR = 0.54; 95% CI: 0.44–0.66; *P* for trend<0.001) and Model 3 (adjusted for all covariates; OR = 0.62; 95% CI: 0.49–0.77; *P* for trend<0.001).

**Table 2:** Associations between PWI and osteoporosis risk.

	Model 1	Model 2	Model3
	(OR, 95% CI, *P*-value)	(OR, 95% CI, *P*-value)	(OR, 95% CI, *P*-value)
PWI (categorical)			
Tertile 1 (≤ 414.50 mL/day)	Reference	Reference	Reference
Tertile 2 (414.50–1024.06 mL/day)	0.73 (0.61, 0.87) <0.001	0.66 (0.54, 0.80) <0.001	0.70 (0.57, 0.86) <0.001
Tertile 3 (> 1024.06 mL/day)	0.56 (0.47, 0.68) <0.001	0.54 (0.44, 0.66) <0.001	0.62 (0.49, 0.77) <0.001
*P* for trend	<0.001	<0.001	<0.001

OR, odds ratio; CI, confidence interval; PWI, plain water intake.

### Subgroup analyses

It was observed that after adjusting for all covariates, a higher PWI was linked to a decreased risk of osteoporosis (OR = 0.92; 95% CI: 0.86–0.98; *p* = 0.008, Table 3). Subgroup analyses were performed to examine whether this relationship varied across the different characteristics of the participants. No significant interactions were identified in the subgroup analyses for age, gender, race, BMI, diabetic history, hypertension status, smoking history, consumption of prednisone or cortisone, or moderate or strenuous activity (all *P* for interaction>0.05).

**Table 3:** Subgroup analyses between PWI (every 500 mL/day) and osteoporosis risk.

Feature		Osteoporosis	*P* for interaction
	*N*	Odds ratio (95% confidence interval), *P*-value	
Total	6,686	0.92 (0.86, 0.98) 0.008	
Age			0.531
< 65	3,550	0.94 (0.85, 1.03) 0.179	
≥ 65	3,136	0.90 (0.83, 0.98) 0.013	
Gender			0.773
Male	3,411	0.90 (0.79, 1.02) 0.111	
Post-menopausal female	2,952	0.93 (0.86, 1.00) 0.041	
Non-menopausal female	323	0.82 (0.56, 1.19) 0.298	
Race			0.349
Hispanic	1,445	0.95 (0.82, 1.10) 0.469	
Non-Hispanic White	3,474	0.90 (0.83, 0.98) 0.014	
Non-Hispanic Black	1,261	0.75 (0.57, 0.98) 0.035	
Other	506	0.99 (0.82, 1.20) 0.925	
Body mass index			0.170
< 30	4,200	0.94 (0.87, 1.01) 0.080	
≥ 30	2,486	0.85 (0.74, 0.97) 0.016	
Diabetic history			0.911
Yes	1,276	0.91 (0.79, 1.05) 0.212	
No	5,162	0.92 (0.86, 0.99) 0.019	
Borderline	248	0.83 (0.52, 1.32) 0.430	
Hypertension status			0.171
Yes	3,581	0.95 (0.88, 1.04) 0.261	
No	3,105	0.88 (0.80, 0.96) 0.006	
Smoking history			0.052
Yes	3,380	0.97 (0.89, 1.05) 0.444	
No	3,306	0.86 (0.78, 0.94) 0.001	
Consumption of prednisone or cortisone			0.983
Yes	417	0.91 (0.73, 1.15) 0.434	
No	6,269	0.91 (0.85, 0.97) 0.006	
Moderate or strenuous activity			0.313
Yes	2,912	0.95 (0.86, 1.05) 0.361	
No	3,774	0.89 (0.83, 0.97) 0.007	

### Smooth curve fitting and threshold effect analysis

**Figure 2:**
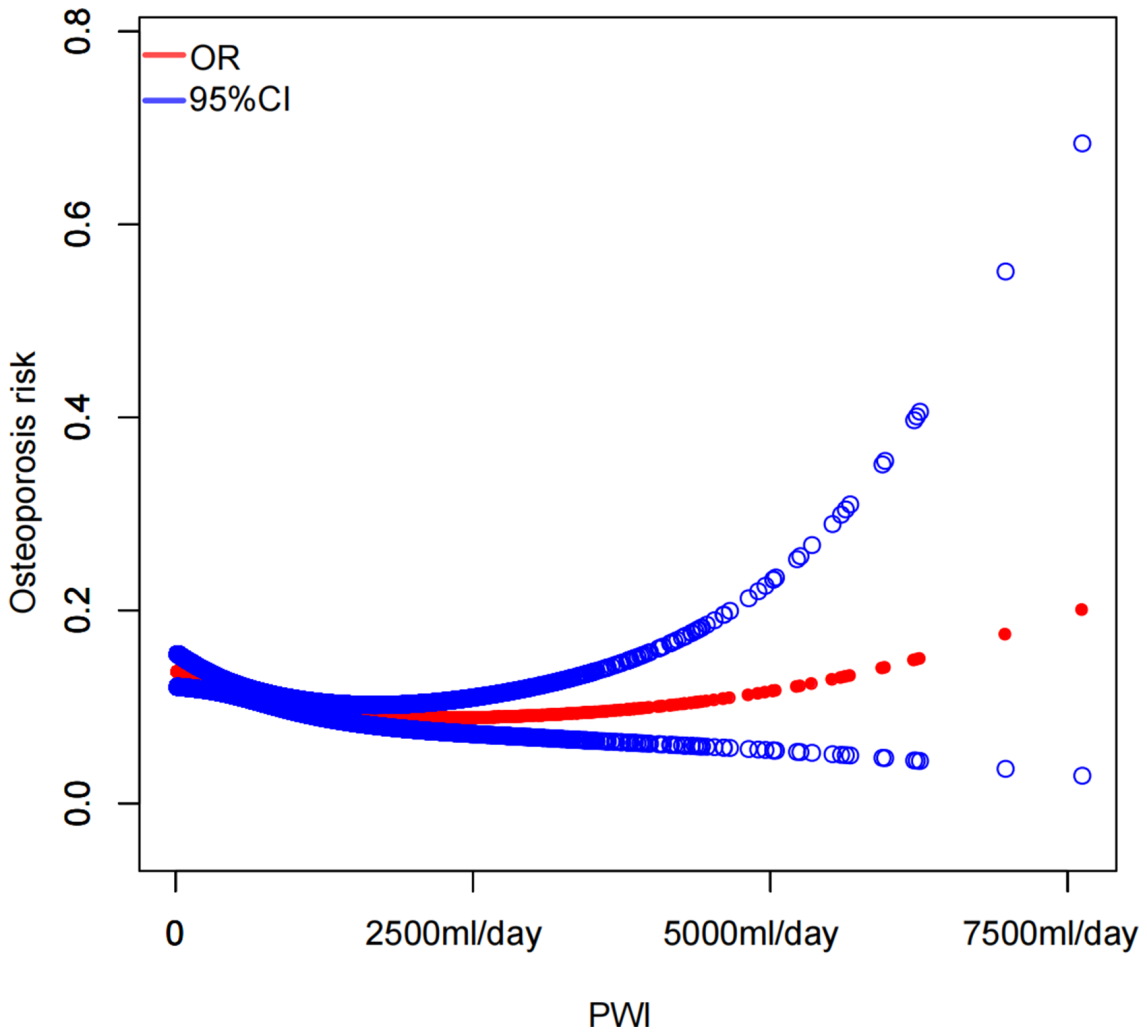
Smooth curve fitting illustrated a nonlinear association between PWI and the risk of osteoporosis.

Smooth curve fitting demonstrated a nonlinear relationship between PWI and osteoporosis risk (Figure 2). The outcomes of the threshold effect analysis are displayed in Table 4. The inflection point, which was identified utilizing a two-piecewise linear regression model, was 2.44 (1,220 mL/day). When PWI was less than 1,220 mL/day, there was a significant negative connection between PWI and osteoporosis risk (OR = 0.79; 95% CI: 0.70–0.89; *p* < 0.001), indicating that osteoporosis risk decreased by 21% for every 500 mL/day increase in PWI. However, that association was not significant when PWI was greater than 1,220 mL/day (OR = 1.06; 95% CI: 0.95–1.17; *p* = 0.288), demonstrating that increasing PWI beyond 1,220 mL/day did not further significantly reduce the risk of osteoporosis.

**Table 4:** Threshold effect analysis of PWI (every 500 mL/day) on osteoporosis utilizing a two-piecewise linear regression model.

Outcome	Osteoporosis (OR, 95% CI, *P*-value)
Fitting by standard linear model	0.92 (0.86, 0.98) 0.008
Fitting by two-piecewise linear model	
Inflection point	2.44 (1,220 mL/day)
< 2.44 (*N* = 4,953)	0.79 (0.70, 0.89) <0.001
> 2.44 (*N* = 1733)	1.06 (0.95, 1.17) 0.288
Logarithmic likelihood ratio test *P*-value	0.002

OR, odds ratio; CI, confidence interval.

## Discussion

In this cross-sectional study with 6,686 participants, the three distinct models indicated that individuals in the greatest PWI tertile had a significantly decreased risk of osteoporosis in contrast to those in the lowest tertile. After adjusting for all covariates, a higher PWI was linked to a decreased risk of osteoporosis. Subgroup analyses exhibited that this trend remained consistent across different population settings. Furthermore, smooth curve fitting and threshold effect analysis indicated that when PWI was less than 1,220 mL/day, a greater PWI was connected with a diminished osteoporosis risk, although an increase in PWI did not further significantly reduce osteoporosis risk when PWI was more than 1,220 mL/day. Notably, 4,953 participants (74.08% of the sample) reported a PWI of less than 1,220 mL/day. Many older US adults and those in middle age may ignore the importance of PWI. Therefore, our investigation is of high significance in the field of public health.

Dietary nutrients are essential for life and serve as the foundation for numerous metabolic processes. A diet rich in balanced nutrients is acknowledged as a preventive measure against osteoporosis, and the impact of nutrition on bone health has garnered growing interest (24). Many dietary nutrients, especially dietary micronutrients, including calcium, vitamin C, iron, potassium, magnesium, phosphorus, and vitamin D, may be strongly associated with osteoporosis (25–32). For example, Lee et al. (33) reported that bone mass might be improved by increasing calcium consumption and maintaining a high dietary calcium/phosphorus ratio. Liu et al. (27) reported that moderate rises in iron consumption were linked with a diminished osteoporosis risk in females. In addition, protein is necessary to maintain bone health and lower daily protein consumption may be connected with a greater risk of osteoporosis (34–36). A cross-sectional study of 4,707 participants revealed that elderly people and those in middle age in the US have an increased risk of osteoporosis when their daily dietary protein consumption is reduced (37). Importantly, fatty acids are one of the components of fat, and consuming fatty acids could be good for bone health (38–40). Fang et al. (38) reported that saturated, monounsaturated, and polyunsaturated fatty acids intake was positively connected with overall BMD among people aged 20 to 59 years. Notably, a cross-sectional study of 4,447 participants revealed that increased carbohydrate consumption was linked to decreased BMD (41). Recently, dietary fiber has been found to have potential benefits for bone health (42–44). Zhang et al. (44) conducted a cross-sectional study with 2,829 individuals and revealed that postmenopausal females with a dietary ratio of carbohydrate/fiber greater than 17.09 have a greater osteoporosis risk, while increased dietary fiber consumption is connected with a diminished risk of osteoporosis. Water is the richest nutrient in the diet, and plain water is the most affordable and accessible source of water consumed in daily life. Nevertheless, the link between PWI and osteoporosis risk has rarely been investigated.

Adequate PWI is crucial for proper body function (45, 46). Prior investigations have discovered the connection between PWI and a variety of diseases or metabolic disorders (47–53). For instance, Li et al. (47) conducted a cross-sectional study of 5,882 individuals and reported that PWI was inversely linked to the risk of periodontitis in those in middle age and older adults in the US. Another cross-sectional study of 16,434 individuals demonstrated that a greater PWI was independently linked with less afresh diagnosed nonalcoholic fatty liver disease in men but not in females (48). Furthermore, Pan et al. (49) performed a 5-year cohort study of 3,200 participants and discovered that a PWI over 4 cups a day was connected with a decreased risk of developing new-onset overweight for people with normal body weight. Most importantly, Lee et al. (50) drew conclusions from a cross-sectional study of 112,250 participants that there was a significant connection between lower PWI and increased risk of self-reported depression or suicidality. This research revealed that, among older US adults and those in middle age, a greater PWI was connected with a moderately diminished risk of osteoporosis. Managing PWI may decrease the osteoporosis risk.

To explain the connection between PWI and osteoporosis risk, we propose several potential mechanisms. Initially, a greater PWI was related to healthier dietary patterns described by greater intake of vegetables, fruits, and dairy products with low and reduced fat (54, 55). Thus, plain water is considered a possible dietary component that could improve dietary micronutrient profiles (56). Dietary micronutrients, including iron, phosphorus, magnesium, calcium, vitamin C, potassium, and vitamin D, may be closely related to osteoporosis. Therefore, a greater PWI may protect bone health through healthier dietary patterns associated with moderately increased intake of certain dietary micronutrients. Second, people with greater PWI were more likely to reduce their sugar-sweetened beverages intake (57). A high intake of sugar-sweetened beverages may reduce BMD (58). As a result, PWI may enhance bone health by decreasing sugar-sweetened beverages consumption. In addition, there were variations in the gut microbiota between people who drank more water and those who consumed less water (59). The gut microbiota can participate in preserving bone balance and protecting against osteoporosis development (60). Consequently, greater PWI may help individuals maintain their bone health through changes in the gut microbiota. Finally, increased daily PWI was shown to decrease the blood urea nitrogen concentration and inhibit the decrease in the estimated glomerular filtration rate (61). The osteoporosis risk was elevated in individuals with a decreased estimated glomerular filtration rate (62). Therefore, a greater PWI may help preserve bone health by inhibiting a reduction in the estimated glomerular filtration rate. Notably, these mechanisms are speculative, and we intend to conduct more research in the future to verify the underlying mechanism(s).

In our study, the ORs between PWI and risk of osteoporosis for all subgroups were < 1. Notably, the association between PWI and osteoporosis risk was significant (*p* < 0.05) in certain subgroups such as post-menopausal female, Non-Hispanic White, or Non-Hispanic Black, whereas it was not significant (*p* > 0.05) in male, non-menopausal female, or any other races. However, larger *p*-values should not be interpreted as indicating no association or no effect: absence of evidence is not evidence of absence (63, 64). In addition, Hodzic-Santor et al. (65) reported that studies with smaller samples are more likely to have larger *p*-values, and studies with larger samples are more likely to have smaller *p*-values. The small number of participants in certain subgroups is a possible reason why the association was not significant.

Our investigation has multiple strengths. First, this study is the first investigation of the correlation between PWI and osteoporosis risk in elderly individuals and those who are middle age in the US. Second, we utilized nationally representative data, which greatly increased the sample size. Third, to ensure the reliability of our outcomes, we adjusted for confounders as much as possible. Finally, we enhanced the robustness of the data analysis by treating each 500 mL/day PWI as a unit and dividing participants into three PWI tertile groups. However, several limitations exist in our study. First, participants could be from different parts of the US, where the chemical composition of the soil, and therefore the water varies. Second, due to data source restrictions, we failed to additionally validate the outcomes using additional NHANES cycles. Third, the 24-h PWI of each participant was determined based on interviews, which may have led to recall bias. Finally, owing to the cross-sectional nature of this study, a causative association between PWI and osteoporosis risk could not be determined. Additional prospective and experimental investigation is necessary in the future to validate the causal link between PWI and osteoporosis risk and to elucidate the underlying processes.

## Conclusion

The findings of our study suggested that among middle-aged and elderly people in the US, a greater PWI was connected with a moderately lower osteoporosis risk. Managing PWI might diminish osteoporosis risk.

## Data Availability

Publicly available datasets were analyzed in this study. This data can be found here: National Health and Nutrition Examination Surveys database (https://www.cdc.gov/nchs/nhanes/).
